# Development and Event-specific Detection of Transgenic Glyphosate-resistant Rice Expressing the *G2-EPSPS* Gene

**DOI:** 10.3389/fpls.2017.00885

**Published:** 2017-05-30

**Authors:** Yufeng Dong, Xi Jin, Qiaoling Tang, Xin Zhang, Jiangtao Yang, Xiaojing Liu, Junfeng Cai, Xiaobing Zhang, Xujing Wang, Zhixing Wang

**Affiliations:** ^1^Biotechnology Research Institute, Chinese Academy of Agricultural SciencesBeijing, China; ^2^Department of Biochemistry, Baoding UniversityBaoding, China; ^3^Biology Institute, Hebei Academy of SciencesShijiazhuang, China

**Keywords:** *G2-EPSPS*, 5-enolpyruvylshikimate-3-phosphate, glyphosate, event-specific PCR, rice

## Abstract

Glyphosate is a widely used herbicide, due to its broad spectrum, low cost, low toxicity, high efficiency, and non-selective characteristics. Rice farmers rarely use glyphosate as a herbicide, because the crop is sensitive to this chemical. The development of transgenic glyphosate-tolerant rice could greatly improve the economics of rice production. Here, we transformed the *Pseudomonas fluorescens* G2 5-enolpyruvyl shikimate-3-phosphate synthase (EPSPS) gene *G2-EPSPS,* which conferred tolerance to glyphosate herbicide into a widely used *japonica* rice cultivar, Zhonghua 11 (ZH11), to develop two highly glyphosate-tolerant transgenic rice lines, G2-6 and G2-7, with one exogenous gene integration. Seed germination tests and glyphosate-tolerance assays of plants grown in a greenhouse showed that the two transgenic lines could greatly improve glyphosate-tolerance compared with the wild-type; The glyphosate-tolerance field test indicated that both transgenic lines could grow at concentrations of 20,000 ppm glyphosate, which is more than 20-times the recommended concentration in the field. Isolation of the flanking sequence of transgenic rice G2-6 indicated that the 5′-terminal of T-DNA was inserted into chromosome 8 of the rice genome. An event-specific PCR test system was established and the limit of detection of the primers reached five copies. Overall, the *G2-EPSPS* gene significantly improved glyphosate-tolerance in transgenic rice; furthermore, it is a useful candidate gene for the future development of commercial transgenic rice.

## Introduction

Rice is one of the four most commonly farmed arable crops (Ray et al., 2013) with about 90% of world production being grown and consumed in Asia (Singh et al., 2016). However, increased rice production is hampered by factors such as decreasing availability of arable land, lack of labor, and water scarcity (Nguyen and Ferrero, 2006). To meet these challenges, there has been a shift from puddled transplanted rice (PTR) to dry direct-seeded rice (DSR) (Farooq et al., 2011). The major challenge for DSR is weed management. Traditional methods of weed control consist predominantly of pulling by hand, or hoeing. In contrast, use of herbicides is more efficient, economical, and labor-saving (Singh et al., 2016).

Glyphosate is a non-selective herbicide first produced by Monsanto in 1970. Its mode of action is to disrupt aromatic amino acid synthesis by inhibiting the enzyme 5-enolpyruvylshikimate-3-phosphate synthase (EPSPS) of the shikimate pathway, thereby controlling the vast majority of weeds. Its qualities of being site-specific, having low toxicity against humans and in the environment, being low-cost, and being broad-spectrum, make glyphosate one of the world’s most commonly used herbicides (Williams et al., 2000; Duke and Powles, 2008). Despite these qualities, glyphosate is rarely used in paddy cultivation because it is harmful to rice. Therefore, the development of glyphosate-tolerant rice cultivars will benefit farmers by reducing labor, water, and energy consumption, which in turn will improve the economics of rice production.

There are two ways to obtain glyphosate-resistant crops: introducing a mutant allele encoding a less-sensitive bacterial enzyme (Stalker et al., 1985), or the overproduction of EPSPS enzyme (Shah et al., 1986; Goldsbrough et al., 1990). In the development of glyphosate-resistant rice, studies on the following genes have taken place: *G6* gene (Zhao et al., 2011), *MdEPSPS* mutant (Tian et al., 2013), *OsEPSPS* mutant (Chandrasekhar et al., 2014), *CP4-EPSPS* (Chhapekar et al., 2015), *VvEPSPS* mutant (Tian et al., 2015), *AroAJ.sp* (Yi et al., 2016), and *I. variabilis-EPSPS^∗^* (*Cui et al., 2016*). The ability of each of these genes to enhance the resistance of rice to glyphosate is not the same. In fact, among these genes, only the *CP4-EPSPS* gene has been widely used for developing commercial glyphosate-tolerant crops; furthermore, the *mepsps* and *2mepsps* genes were only used on a few types of GM plants^1^. The single source of the *EPSPS* gene is probably the cause of the reduction in herbicide tolerance; the latter has become the main concern of those involved in field management programs (Tian et al., 2015). For these reasons, exploration of the application of the glyphosate-tolerance gene to a variety of crops has great agricultural significance.

The *G2-EPSPS* gene (GenBank Accession No.: EF155478) encoding a highly glyphosate-resistant EPSPS protein was identified from *Pseudomonas fluorescens* strain G2, which was isolated from a storage area with a history of glyphosate pollution (Zhu et al., 2003). Dun et al. (2007) transferred the *G2-EPSPS* gene into tobacco and the transgenic tobacco plants were capable of achieving normal growth at 1% glyphosate. When the *G2-EPSPS* was transferred into cotton by Zhang et al. (2016), the herbicide-resistant cotton cultivar BG2-7 was created, in which glyphosate resistance reached 8000 ppm. Guo et al. (2015) co-expressed the *G2-EPSPS* and *GAT* genes in soybean, and no typical symptoms of glyphosate poisoning were observed in the transgenic soybean at 900–3600 g a.e. ha^-1^. Liu et al. (2015) transferred the same gene into maize; when the transgenic maize was subsequently sprayed with 4 g L^-1^ glyphosate at a dose of 2.46 kg ha^-1^, the G2-EPSPS conferred good resistance even after 3 days. The above results therefore indicate that *G2-EPSPS* is a good candidate gene for improving glyphosate resistance in a variety crops. However, its effects in rice have not yet been reported; whether it can confer a comparable degree of glyphosate resistance in this crop is unknown.

In the current study, the *G2-EPSPS* gene was transferred into rice plants to produce transgenic rice with good resistance to glyphosate. Furthermore, the effects of different concentrations of glyphosate on the growth of transgenic rice were studied. The insertion site of T-DNA was determined by cloning the left flanking sequence of G2-6 transgenic rice. An event-specific PCR detection method was developed, using specifically designed primers, to detect line G2-6 and to establish the lower limit of detection.

## Materials and Methods

### Construction of Plant Transformation Vector and Rice Transformation

**FIGURE 1:**
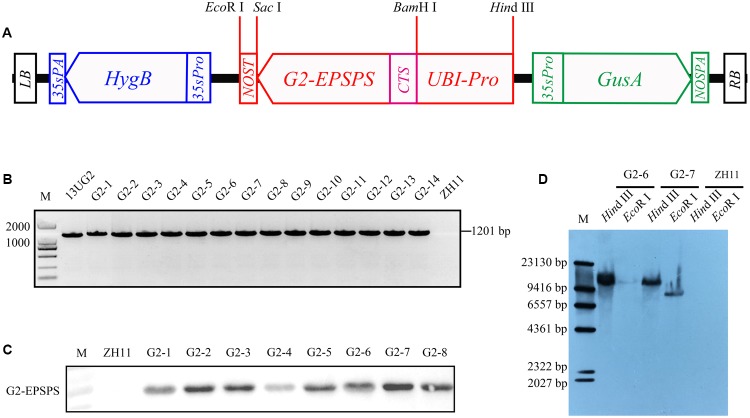
Transformation and detection of transgenic rice. **(A)** Diagrams of the plasmid constructs *UBI::CTS::G2-EPSPS*, containing the *CTS::G2-EPSPS* coding region with a *nos* terminator under control of the *UBI* promoter. **(B)** The transgenic-line-specific PCR was implemented using *G2-EPSPS* gene-specific primers. The vector *13UG2* was used as the positive control (lane *13UG2*), and ZH11 DNA was used as the negative control (lane ZH11). **(C)** Expression analysis of the G2-EPSPS protein in the shoot of ZH11 (designated as negative control) and eight *UBI::CTS::G2-EPSPS* T0 transgenic lines (G2-1, G2-2, G2-3, G2-4, G2-5, G2-6, G2-7, G2-8). **(D)** Transgenic rice genome DNA was digested with restriction endonuclease *Hin*dIII and *Eco*RI. The DIG-labeled *G2-EPSPS* gene probe was used to detect T-DNA copy number in transgenic lines.

The *Chrysanthemum x morifolium* ribulose-1,5-bisphosphate carboxylase small subunit chloroplast signal peptide gene *CTS* was amplified from Impactvector 1.4 by PCR with primers CTS-1/CTS-2, and the *G2-EPSPS* gene was cloned from *Pseudomonas fluorescens* by PCR with primers G2-1/G2-2. The *CTS::G2-EPSPS* fusion gene was amplified by overlap PCR, using primers containing a *Bam*H I restriction site at the 5′-end of forward primer (*Bam*H I-CTS-1: GGATCCggatcctatggcctcgatctcttcc) and a *Sac* I restriction site at the 5′-end of reverse primer (*Sac* I-G2-2: GAGCTCgagctctcagtcgtttaggtgaacg), respectively. The PCR conditions were five cycles of 95°C for 25 s, 65°C for 25 s, and then 72°C for 70 s with no primers. Subsequently, primers were added into the PCR tube, and the cycle was run at 95°C for 5 min, followed by 29 cycles at 95°C for 25 s, 57°C for 25 s, and 72°C for 70 min; and finally 72°C for 5 min. The UBI promoter from pHAC25 was sub-cloned into pBI121 with *Hin*d III and *Bam*H I restriction sites to generate the pUBI121 vector. The fusion gene *CTS::G2-EPSPS* was inserted into the pUBI121 vector by *Bam*H I and *Sac* I to generate the *pUBI121::CTS::G2-EPSPS* vector. Subsequently, the new vector was digested by *Hin*d III and *Eco*R I and cloned into the multiple cloning site of pCAMBIA1301 to generate the vector 13UG2 (**Figure 1A**). The vector was introduced into *Agrobacterium tumefaciens* EHA105. Transformation of the *Japonica* cultivar ZH11 was performed based on the method reported by Hiei et al. (1994).

### PCR Analysis of Transgenic Rice

Rice genomic DNA was isolated from hygromycin resistance rice leaves using the modified CTAB method (Porebski et al., 1997) and amplified with the primers G2-F and G2-R1 (Supplementary Table S1). PCR was carried out with 50 ng of rice genomic DNA, 10 μl of 2× PCR mix (2× EasyTaq PCR SuperMix, Transgene, Beijing, China), and 0.5 μl each of G2-F (10 μM) and G2-R1 (10 μM). The PCR conditions were 95°C for 8 min, followed by 32 cycles of 95°C for 25 s, 53°C for 40 s, and 72°C for 1 min; and finally, 72°C for 5 min.

### Western Blot Analysis

Total protein was extracted from 0.1 g of transgenic rice leaves using a Plant Protein Extraction Kit (CWBIO, CW0885, Beijing, China), and the amount of protein was calculated using the bicinchoninic acid (BCA) method. About 10 μg of protein was denatured at 95°C for 8 min, and then resolved by SDS-PAGE on 12% gels; proteins were then wet-blotted onto a nitrocellulose filter membrane. The G2-EPSPS protein was detected using the primary monoclonal antibody, anti-G2-EPSPS (3 mg/ml, 1:2000 dilution), which was obtained from immune mice using G2-EPSPS protein purified from the prokaryotic expression system. Horseradish peroxidase- (HRP)-labeled goat anti-mouse antibodies were used as the secondary antibody diluted at a ratio of 1:5000, and enhanced chemiluminescence (ECL) was used to detect the blot.

### Southern Blot Analysis

Aliquots of 40 μg purified DNA were digested with *Hind* III and *EcoR* I, respectively. The digested DNA fragments were separated on a 0.8% agarose gel and then blotted onto Hybond^TM^-N^+^ nylon membrane (GE Healthcare UK Limited). The probe was amplified using primers G2-F/G2-R2 (Supplementary Table S1), and labeled with DIG (PCR DIG Probe Synthesis Kit, Roche, Germany). The blot was washed and detected according to the product instructions (DIG High Prime DNA Labeling and Detection Starter KitII, Roche, Germany).

### Plant Material and Growth Conditions

For glyphosate-tolerance studies, seeds from each of the non-transgenic control ZH11 and homozygous transgenic plants (T_3_ generation) were germinated in ultrapure water containing different concentrations of glyphosate at 37°C for 1 day in the dark. To test seed germination in ZH11 and transgenic lines, 40 seeds per dish were grown in 0, 50, and 100 ppm concentrations of glyphosate for 6 days. The glyphosate used in the report was Roundup, which contains 41% isopropyl amine salt of glyphosate (Monsanto, Malaysia), and 1000 ppm glyphosate, equivalent to 0.25% (v/v) Roundup.

For experimental analyses, seeds from the non-transgenic control ZH11 and transgenic lines (T_3_ generation) were germinated in ultrapure water at 37°C for 1 day in the dark, and then transferred into soil in the greenhouse under controlled conditions. About 30 seedlings of ZH11 per pot were grown for 4 weeks, and then sprayed with 0, 1000, 2000, or 3000 ppm of glyphosate to test for lethal concentrations. The two homozygous transgenic lines were each sprayed with 0, 3000, 5000, 8000, 10,000, 12,000, 15,000, or 20,000 ppm of glyphosate at the tillering stage. Twelve days later, all treated plants were photographed and shoot height was measured.

For the glyphosate-tolerance assay, seeds of ZH11 and transgenic lines (T_4_ generation) were transferred to 1/2 Yoshida hydroponic culture with 1 mM NH_4_NO_3_ (pH 5.5) containing different concentrations of glyphosate for 10 days, and the nutrient solution was changed every day. ZH11 was grown in 0, 0.01, 0.1, 0.2, 0.5, 1, 4, and 16 ppm glyphosate; transgenic plants were grown in 0, 5, 10, 25, 50, 100, 200, 800 ppm glyphosate for 10 days. The seedlings were kept in a growth chamber [28°C; 14-h/10-h light/dark photoperiod; 90% humidity; photon flux density, 500 μmol/m^2^/s photosynthetically active radiation (PAR)]. After 10 days, all the plants were collected. Plant height data at each glyphosate concentration were expressed as relative height, by comparing the mean height of each transgenic line at each concentration with its mean height at 0 ppm glyphosate. The relative heights at different concentrations could be fitted to a sigmoidal logistic model to produce dose-response curves (Seefeldt et al., 1995; Cui et al., 2016).

### RNA Isolation and Quantitative RT-PCR Analysis

Total RNA was extracted from control and transgenic plant shoot tissues using RNAiso Plus (Takara Biotechnology, Dalian, China). Approximately 1.5 μg of total RNA was used to synthesize first-strand cDNA using a PrimeScript^TM^ RT reagent Kit with gDNA Eraser (Takara). In the transgenic plant expression analyses, transcript levels were normalized to those of the *OsUBQ5* gene, and *G2-EPSPS* gene expression level was detected by the primers G2-151F/G2-151R (Supplementary Table S1). Real-time PCR was carried out in 7500 Real-Time PCR System (Applied Biosystems, United States) in 20 μl reaction mixtures containing 2 μl cDNA sample (1:20 dilution), 0.4 μl forward primers (10 μm), 0.4 μl reverse primers (10 μm), 0.4 μl Dye II and 10 μl SYBR Green Real-time PCR Mix (Takara Biotechnology, Dalian, China), the PCR conduction was at 95°C for 15 s, followed by 40 cycles of 95°C for 5 s and 60°C for 34 s.

### Isolation of the Flanking Sequence of T-DNA

The GenomeWalker^TM^ Universal Kit (Clontech, Takara) was used to isolate the flanking sequence of T-DNA; 25 μg of transgenic rice genomic DNA was digested with restriction endonucleases *Dra*I, *Eco*R V, *Pvu*II, and *Stu*I, respectively, and the adaptor supplied in the kit was added. The specific primer GPS1/2 was designed and combined with the left boundary of the T-DNA. PCR was conducted according to the kit protocol. The sequence was analyzed using BLAST software in the NCBI database to search for the integration feature of T-DNA on the rice genome using the Rice Genome Annotation Project database^2^.

### Establishment of Event-Specific PCR and Genomic DNA Detection Limit for Transgenic Plants

For integration event-specific PCR detection analysis, the multiplex PCR method was used. The event-specific primers G_2_-OsF/G_2_-GR were designed according to the 5′-flanking sequence of the T-DNA of G2-6 transgenic rice and the sequence near the left-border of 13UG2. Additionally, *OsSPS* (sucrose-phosphate synthase 1, LOC_Os01g69030) specific primers SPSF/SPSR were designed, as a positive control in the multiplex amplification system (Supplementary Table S1). Multiplex PCR was conducted using genomic DNA isolated from transgenic rice with different integration events as the template. The PCR conditions were 95°C for 8 min, followed by 32 cycles at 95°C for 25 s, 54°C for 25 s, and 72°C for 30 s; and then extension at 72°C for 5 min.

To determine the detection limit for transgenic plants, the concentration of template DNA isolated from G2-6 was measured and diluted to 5, 10, 20, 40, 80, 160, and 320 copies, respectively. Then, to identify the detection limit using the integration-event-specific PCR system, a negative control was performed without using the DNA template.

## Results

### Production and Identification of Transgenic Rice

A total of 46 independent transgenic plants were obtained. Rice genomic DNA was isolated from T_0_ generation transgenic plants, and PCR amplification was performed with *G2-EPSPS* specific primers G2-F/G2-R1. A 1201 bp fragment was produced in the transgenic rice, whereas PCR products could not be amplified in ZH11, showing that the target gene was successfully integrated (**Figure 1B**). To determine G2-EPSPS protein expression in transgenic rice, proteins were extracted from PCR-positive transgenic rice leaves, and all the selected transgenic lines had G2-EPSPS protein expression; here only eight results were listed, as shown in **Figure 1C**. Southern blotting was performed with the T_0_-generation plant DNA. Results showed that G2-6 and G2-7 contained a single copy of the *G2-EPSPS* gene (**Figure 1D**). The *G2-EPSPS* gene had similar expression levels in leaves of both G2-6 and G2-7 (**Supplementary Figure S1**).

### Glyphosate-Tolerance of Germinated Homozygous Transgenic Lines G2-6 and G2-7

**FIGURE 2:**
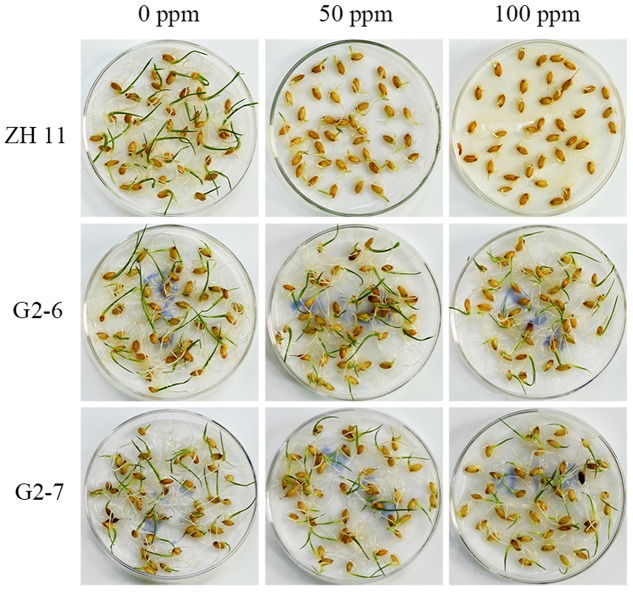
Germination of transgenic plant seeds under different concentrations of glyphosate. Sprouting of homozygous transgenic lines (G2-6, G2-7) on ultrapure water containing 0, 50, or 100 ppm glyphosate for 6 days.

Glyphosate can affect seed germination, and seeds are usually inhibited and bleached under glyphosate stress (Dun et al., 2007). To investigate the agronomic performance of the two transgenic lines, seeds and soil culture seedlings were treated with different concentrations of glyphosate. Seeds from ZH11, G2-6, and G2-7 were germinated in ultrapure water with 0, 50, and 100 ppm glyphosate for 6 days (**Figure 2**). The results showed that G2-6 and G2-7 could germinate well at 50 ppm and 100 ppm concentration of glyphosate; the radicle also grew normally at 100 ppm glyphosate, whereas ZH11 germination was obviously inhibited at 50 ppm glyphosate, and completely inhibited at 100 ppm glyphosate. These findings indicated that transgenic lines were more tolerant to glyphosate than the non-transgenic variety ZH11.

### Glyphosate-Tolerance of Homozygous Transgenic Lines G2-6 and G2-7 in the Field

**FIGURE 3:**
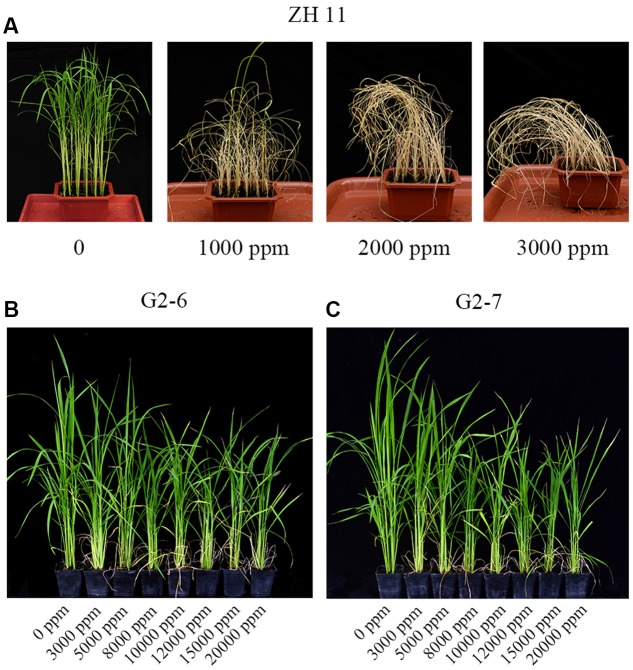
Rice growth phenotype under different concentrations of glyphosate treatment. **(A)** ZH11 grown in the greenhouse for 4 weeks, and photographed 12 days after treatment with 0, 1000, 2000, or 3000 ppm glyphosate. **(B,C)** transgenic plants G2-6 and G2-7 grown in the greenhouse at the tillering stage, and photographed 12 days after treatment with 0, 3000, 5000, 8000, 10,000, 12,000, 15,000, or 20,000 ppm glyphosate.

Four-weeks-old seedlings of the control ZH11 were treated with 0, 1000, 2000, and 3000 ppm glyphosate. After 12 days, all treated ZH11 plants were dead, leaving only the 0 ppm glyphosate treatment group alive (**Figure 3A**). This result indicated that in the field, 1000 ppm glyphosate is a lethal concentration for rice.

To determine the tolerance of the transgenic cultivars G2-6 and G2-7 to much higher glyphosate concentrations, they were treated with 0, 3000, 5000, 8000, 10,000, 12,000, 15,000, or 20,000 ppm glyphosate in the field at the tillering stage. After 12 days, plants under all treatments were fully expanded and green, but the shoot heights were different and were therefore used for statistical analysis of the agronomic performance of transgenic lines (**Figures 3B,C**). There were significant differences in plant shoot height between homozygous lines and glyphosate dosages (**Table 1**).

**Table 1:** Agronomic performances of selected transgenic lines under different glyphosate treatments.

Homozygous lines	Glyphosate dosage (ppm)	Shoot height (cm)
G2-6	0	79.16 ± 1.53a
	3000	64.96 ± 1.81b
	5000	59.96 ± 1.45b
	8000	50.02 ± 1.64c
	10,000	48.32 ± 4.62c
	12,000	48.1 ± 6.36c
	15,000	45.91 ± 1.31c
	20,000	45.09 ± 3.83c
G2-7	0	78.56 ± 1.29a
	3000	67.45 ± 2.09b
	5000	64.35 ± 4.52b
	8000	57.1 ± 3.06c
	10,000	55.6 ± 2.98c
	12,000	56.23 ± 2.98c
	15,000	54.4 ± 2.53c
	20,000	53.26 ± 3.36c
Source of variation		
Homozygous lines (HL)		^∗∗^
Glyphosate dosage (GD)		^∗∗^
HL × GD		ns

*The parameters are shown as average values ( ± standard deviation), which were collected from 40 plants in three replicates for each treatment. Numbers followed by different letters indicate significant differences (*P* < 0.05). ns, not significant (*P* > 0.05). ^∗∗^ Significant at *P* < 0.01*.

When treated with 3000 ppm or higher glyphosate, shoot heights of both transgenic lines were significantly decreased compared with untreated group, whereas at 8000, 10,000, 12,000, 15,000, and 20,000 ppm of glyphosate, the shoot heights of G2-6 and G2-7 exhibited no significant differences, respectively. Furthermore, shoot height of G2-6 was decreased by 15.3% when compared with G2-7 in the 20,000 glyphosate treatment group. These results indicated that G2-7 may show better agronomic performance than G2-6, although they exhibited similar expression levels. With further cultivation, the differences of shoot height disappeared at the heading stage; all treatments then progressed to the same height as the untreated group, and produced seeds normally.

### Glyphosate-Tolerance of Homozygous Transgenic Lines G2-6 and G2-7 in a Hydroponic System

**FIGURE 4:**
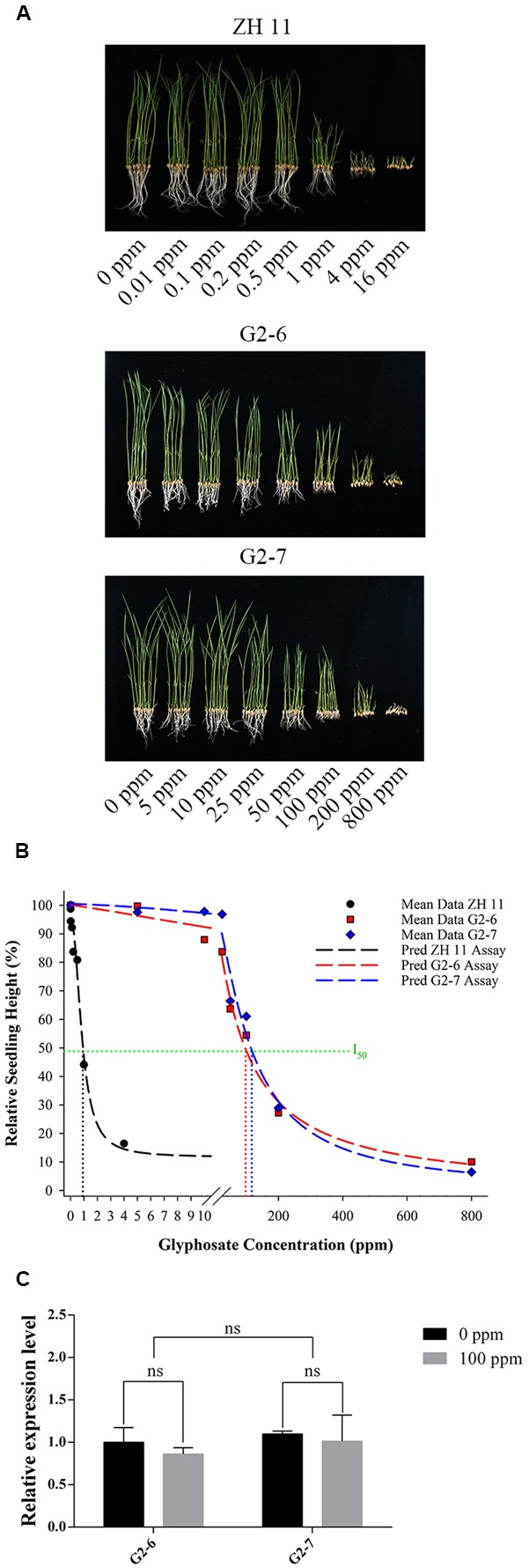
Glyphosate-tolerance assay on hydroponic culture. **(A)** ZH11, G2-6, and G2-7 seedlings were grown in aaa Yoshida hydroponic culture with 1 mM NH_4_NO_3_, containing different concentrations of glyphosate, for 10 days. **(B)** Dose-response curves for ZH11, G2-6, and G2-7 were drawn giving the relative plant height in each line. The predicted logistic equations for ZH11, G2-6, and G2-7 are Y_ZH11_= 11.68 + 83.58/[1 + (X/0.86)^2.18^] [*R*^2^= 0.993, I_50_= 0.93], Y_G2-6_= 0.23 + 99.99/[1 + (X/95.6)^1.09^] [*R*^2^= 0.994, I_50_= 96.4], and Y_G2-7_= 0.06 + 100.5/[1 + (X/113.06)^1.4^] [*R*^2^= 0.989, I_50_= 114.07]. **(C)** Expression level of *G2-EPSPS* gene in the shoots of transgenic lines G2-6 and G2-7 under 0 and 100 ppm glyphosate concentration. ns, not significant (*P* > 0.05).

There were significant differences in plant shoot height and plant fresh weight between homozygous lines, and with glyphosate dosage. In the hydroponic system, increase in shoot height and plant fresh weight of ZH11 could be significantly inhibited at 0.2 ppm glyphosate. The ZH11 seedlings were decomposed at 4 ppm glyphosate concentration, and according to the dose response curve, the relative seedling height was not obviously reduced with increasing glyphosate concentration; the results indicated that ZH11 was completely inhibited by 4 ppm glyphosate (**Figure 4A** and **Table 2**).

**Table 2:** Hydroponic growth phenotype of non-transgenic lines under different glyphosate treatments.

Non-transgenic lines (ZH11)	Glyphosate dosage (ppm)	Shoot height (cm)	Plant weight (g plant^-1^)
	0	15.30 ± 0.24a	0.8 ± 0.04ab


	0.01	14.62 ± 0.22a	0.81 ± 0.01ab


	0.1	14.28 ± 0.84a	0.84 ± 0.05a


	0.2	12.97 ± 0.63b	0.71 ± 0.07b


	0.5	12.52 ± 1.08b	0.62 ± 0.05c


	1	6.83 ± 0.51c	0.45 ± 0.03d


	4	2.55 ± 0.22d	0.16 ± 0.02e


	16	1.65 ± 0.11d	0.1 ± 0.02e

*The parameters are shown as average values (± standard deviation) that were collected from 40 plants in three replicates for each treatment. Numbers followed by different letters indicate significant differences (*P* < 0.05)*.

For G2-6 and G2-7, at increased treatment concentrations of glyphosate, shoot heights were significantly decreased to differing degrees (**Table 3** and **Figure 4A**). For G2-6, shoot height and plant fresh weight was significantly inhibited at 10 ppm glyphosate, whereas for G2-7 the corresponding inhibitory concentration was 50 ppm. For both G2-6 and G2-7, shoot height and plant fresh weight could be completely inhibited at 800 ppm glyphosate; at this application rate the seedlings performed similar to ZH11 when treated with 4 ppm glyphosate.

**Table 3:** Agronomic performances of the selected transgenic lines under different glyphosate treatments.

Homozygous lines	Glyphosate dosage (ppm)	Shoot height (cm)	Plant weight (g plant^-1^)


G2-6	0	16.45 ± 0.22a	0.79 ± 0.01a


	5	16.4 ± 0.68a	0.78 ± 0.05a


	10	14.47 ± 0.05b	0.71 ± 0.01b


	25	13.77 ± 0.14b	0.68 ± 0.04b


	50	10.48 ± 0.71c	0.46 ± 0.02c


	100	8.96 ± 0.55d	0.38 ± 0.02c


	200	4.48 ± 0.4e	0.12 ± 0.05d


	800	1.65 ± 0.24f	0.05 ± 0.03d


G2-7	0	16.8 ± 0.83a	0.84 ± 0.01a


	5	16.4 ± 1.83a	0.89 ± 0.03a


	10	16.44 ± 1.01a	0.85 ± 0.01a


	25	16.28 ± 1.44a	0.82 ± 0.03a
	50	11.16 ± 0.56b	0.52 ± 0.06b
	100	10.26 ± 0.53b	0.46 ± 0.13b
	200	4.86 ± 0.8c	0.1 ± 0.02c
	800	1.09 ± 0.1d	0.03 ± 0.01c
Source of variation			
Homozygous lines (HL)		^∗∗^	^∗∗^
Glyphosate dosage (GD)		^∗∗^	^∗∗^
HL^∗^GD		ns	ns

*The parameters are shown as average values ( ± standard deviation) that were collected from 40 plants in three replicates for each treatment. Numbers followed by different letters indicate significant differences (*P* < 0.05). ns, not significant (*P* > 0.05). ^∗∗^ Significant at *P* < 0.01*.

To investigate the glyphosate-tolerance of the transgenic lines, dose-response curves were created using the relative shoot heights of ZH11, G2-6, and G2-7 under different glyphosate concentrations (**Figure 4B**). The results revealed that the I_50_ for each line was 0.93, 96.4, and 114.07 ppm, respectively. The glyphosate-tolerance of G2-6 and G2-7 was 104- and 123-times that of ZH11, respectively.

To better understand the glyphosate-tolerance phenotype of the transgenic lines, we further analyzed *G2-EPSPS* gene expression levels in the shoots of plants under the 0 and 100 ppm glyphosate treatments. For both G2-6 and G2-7, there was no significant difference in *G2-EPSPS* gene expression level between 0 and 100 ppm glyphosate treatment (**Figure 4C**).

### Establishment of an Event-Specific PCR Detection Method

To analyze the integrity of transgene insertion in the plant genome, we isolated the left-border flanking sequences of T-DNA from the G2-6 transgenic line. Within the obtained 863 bp sequence, there were 612 bp showing 100% similarity to the 13UG2 vector sequence. The 251 bp upstream sequences exhibited 99% similarity to the sequence of *Oryza sativa* chromosome 8 from 23685037 to 23685287. The results indicated that the 5’-terminus of T-DNA was inserted at position 23685287 of the *Oryza sativa* chromosome 8. Further analysis showed that, in G2-6, the distance of the T-DNA integration sites to the upstream protein coding sequence (CDS) was 14 kb and to the downstream protein CDS it was 26 kb.

**FIGURE 5:**
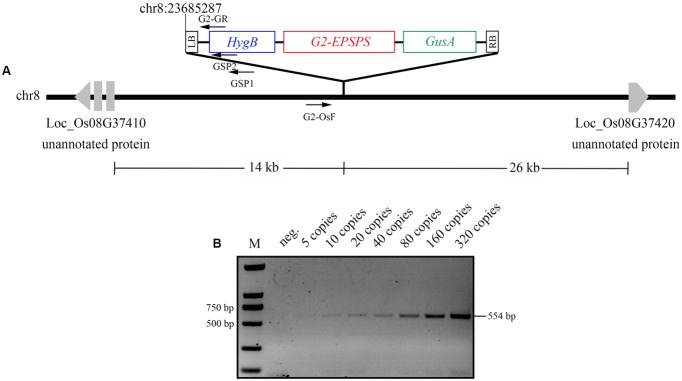
Integration-site analysis and integration-event-specific PCR analysis. **(A)** The T-DNA LB integration site was identified by GenomeWalker PCR with specific primers GSP1, GSP2. The event-specific primers G2-OsF/G2-GR were designed according to the 5′-flanking sequence of the T-DNA of G2-6 transgenic rice and the left-border sequence of 13UG2. **(B)** Identification of the detection limit of transgenic plants by integration-event-specific PCR. Transgenic plant G2-6 template DNA copies were diluted to concentrations of 5, 10, 20, 40, 80, 160, and 320 copies as templates for integration-event-specific PCR.

To develop event-specific PCR detection of G2-6 and ensure credibility of the PCR system, an endogenous reference gene *OsSPS* was selected as the positive control. The specific primers G2-OsF/G2-GR were designed according to the sequence of the rice genome and the left-border region of 13UG2, respectively (**Figure 5A**). A 554 bp fragment could be amplified from G2-6, whereas no PCR product could be amplified from other transgenic lines, ZH11, or the negative control without DNA template. A 151 bp fragment could be amplified from all transgenic lines and the non-transgenic line ZH11, whereas no PCR product could be amplified from the negative control (**Supplementary Figure S2**). The results confirmed that conventional PCR could identify the specific integration events of G2-6 and could therefore be used successfully for detecting transgenic rice G2-6.

### Sensitivity Testing of the Event-Specific PCR System

To test the limits of the event-specific PCR system, the DNA template of transgenic rice G2-6 was diluted to 5, 10, 20, 40, 80, 160, and 320 copies. A weak 554 bp fragment could be amplified from five copies, and with an increase in the template copy number, the brightness increased (**Figure 5B**). The detection limits were acceptable under the regulatory requirements of China.

## Discussion

Since GM crops have increased in popularity over the last decade, concerns about the safety of GM foods among the public have escalated. For the *G2-EPSPS* gene, there has been some research regarding the food safety of GM maize. According to Zhu et al. (2013), the allergenic and toxicity analysis demonstrated that no amino acid sequence similarities were observed between G2-EPSPS enzymes and known allergenic and toxic proteins. The G2-EPSPS enzyme is readily degraded in simulated gastric/intestinal fluid within 15 s. In addition, a 90-day feeding trial in Sprague-Dawley (SD) rats of *G2-EPSPS* GM maize indicated that GM glyphosate-tolerant maize was as safe and nutritious as conventional maize. Further, a 90-day feeding study of the stacked trait GM maize GH5112E-117C (obtained from Beijing Origin Seed Technology, Inc., China) with *Cry1Ah* and *G2-EPSPS* gene was conducted by Han et al. (2016), and the results were similar to those of Zhu et al. (2013). These risk assessments of GM maize indicate that there is little potential risk to food safety through introducing the G2-EPSPS enzyme into food or feed (Zhu et al., 2013).

Research into rice tolerance to the herbicide glyphosate is mainly focused on the several established strains of glyphosate-tolerant rice. Previous results show that all transgenic rice with the *G6* gene survived under 8 g/L, 100 ml/m^2^ glyphosate *(*Zhao et al., 2011); the resistance level of *MdEPSPS _mutant_* transgenic plants is up to 2.5% at 10 L/ha (Tian et al., 2013); and that *Os-mEPSPS* and *ASAL* genes are co-expressed in rice. The leaves of transgenic seedlings remain fully expanded and green after being grown for 24 h in 4 mM glyphosate solution and subsequently transferred to a hydroponic solution without glyphosate (Chandrasekhar et al., 2014). Furthermore, *CP4-EPSPS* transgenic rice can tolerate up to 1% of commercial Roundup, which is five times the dose used to kill weeds under field conditions (Chhapekar et al., 2015). The *VvEPSPS* mutant gene can tolerate up to 1 L/ha glyphosate (Tian et al., 2015), and *AroA*_J.sp_ allows transgenic rice to tolerate up to 3360 g/ha glyphosate, a dosage that is fourfold the recommended agricultural application level (Yi et al., 2016). *I. variabilis-EPSPS* transgenic rice can sprout on a medium containing 2160 mg L^-1^ and *G2-EPSPS* candidate transgenic lines are not affected even when the glyphosate dosage increases to 8400 g ha^-1^ (Cui et al., 2016). Since its isolation, the *G2-EPSPS* gene has been shown to confer good glyphosate resistance in many plants. The genetic glyphosate-tolerant rice containing the *G2-EPSPS* gene that we generated in the current study can tolerate a concentration of 20,000 ppm glyphosate [equivalent to 5% (v/v) Roundup (isopropyl amine salt at 41% w/v), 20 g^-1^ or 20 mM]; in contrast, 1000 ppm glyphosate was sufficient to kill non-transgenic rice and most weeds. The dosage we used in this report is about 20-fold the recommended concentration. Furthermore, under hydroponic conditions, transgenic rice roots total immersion into the nutrient solution at different concentrations of glyphosate, through to the shoot length measurement and statistical analysis, the two transgenic lines improved the glyphosate resistance by about 100-fold over ZH11. These results indicated that the *G2-EPSPS* gene conferred a high tolerance to glyphosate herbicide in rice, and this is consistent with previous research results on other plants.

It is widely known that the T-DNA insertion may confer some unexpected traits on transgenic plants (Latham et al., 2006). The T-DNA insertional mutagenesis and the unexpected effect from the context gene may lead G2-6 and G2-7 to respond differently to glyphosate. In our research, both G2-6 and G2-7 showed similar expression levels of the integrated *G2-EPSPS* gene, but there were small differences between G2-6 and G2-7; for example, hydroponic cultured G2-7 was significantly inhibited at 50 ppm glyphosate whereas G2-6 was significantly inhibited at 10 ppm, and the G2-7 transgenic line showed greater plant height when sprayed with 8000–20,000 ppm glyphosate in the field. Even though these are small differences, they demonstrated a similar response trend to glyphosate pressure. For example, both G2-6 and G2-7 plant height were inhibited when sprayed with 3000 ppm and 5000 ppm glyphosate, and the inhibition was more significant when the glyphosate concentration was equal to or greater than 8000 ppm. However, plant height was not inhibited significantly when the glyphosate concentration was greater than 8000 ppm. Hydroponic culture using different concentrations of glyphosate showed that G2-6 and G2-7 plant height were both inhibited by 50% relative height at about 100 ppm glyphosate, and the heights of both were completely inhibited at 800 ppm. Thus, the good glyphosate-tolerance presented in G2-6 and G2-7 is conferred by the *G2-EPSPS* gene and not by the specificity of individual transformation events.

Transferred genes must be both stably integrated and expressed, this stability is a prerequisite for commercial use (Mehrotra and Goyal, 2013). The establishment of the transformation-event-specific PCR detection method is important for transgenic breeding and the protection of food, feed, and environmental safety (Holst-Jensen et al., 2012; Lee et al., 2015; Guo et al., 2016). In the current study, an event-specific PCR system for the transformation of transgenic rice G2-6 was established, and each generation of G2-6 transgenic rice was detected by event-specific PCR analysis. The results also showed that the position of the T-DNA remained unchanged, and provided a basis for the evaluation of G2-6 transgenic rice.

## Conclusion

The *G2-EPSPS* gene shows promising potential for commercial application in the improvement of glyphosate-resistant rice.

## Author Contributions

ZW and XW conceived and designed the experiments, YD performed the experiments, and wrote the paper, XJ constructed the plant transformation vector and performed the rice transformation, QT and JC improved the language, YD, QT, XinZ, XiaZ, JY, XL analyzed the data.

## Conflict of Interest Statement

The authors declare that the research was conducted in the absence of any commercial or financial relationships that could be construed as a potential conflict of interest.

## Footnotes

1 http://www.isaaa.org/gmapprovaldatabase/default.asp

2 https://blast.ncbi.nlm.nih.gov/Blast.cgi

## Supplementary Material

FIGURE S1 ***G2-EPSPS* gene expression level in T3 transgenic lines**.

FIGURE S2 **Integration-event-specific PCR of G2-6 transgenic lines**.
