# Supplementary figures and images for: Development and Event-specific Detection of Transgenic Glyphosate-resistant Rice Expressing the G2-EPSPS Gene
Source: Front Plant Sci. 2017 May 30;8:885. doi: 10.3389/fpls.2017.00885 (PMC5447670; doi:10.3389/fpls.2017.00885)

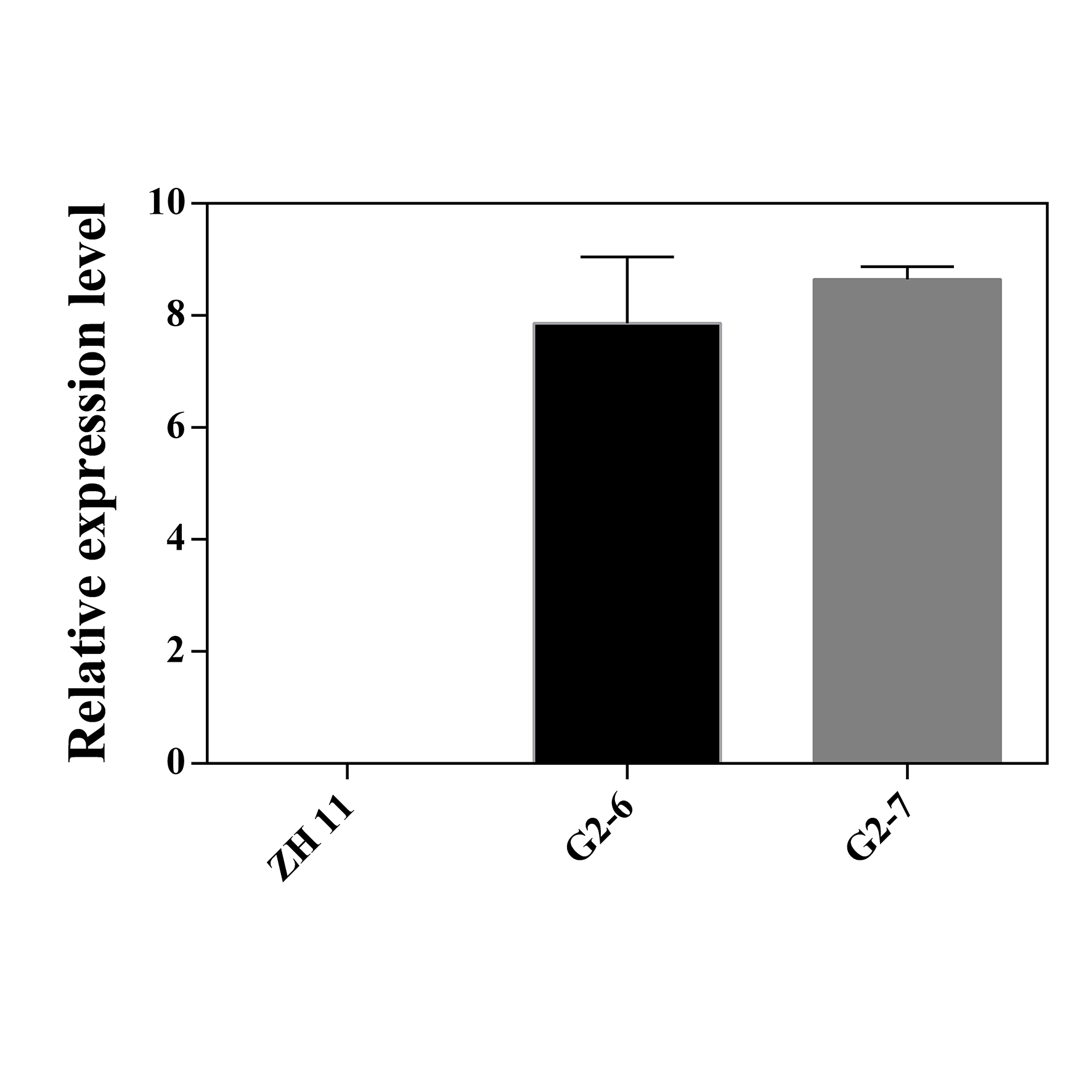

Supplement: FIGURE S1 — G2-EPSPS gene expression level in T3 transgenic lines. [file Image_1.JPEG]

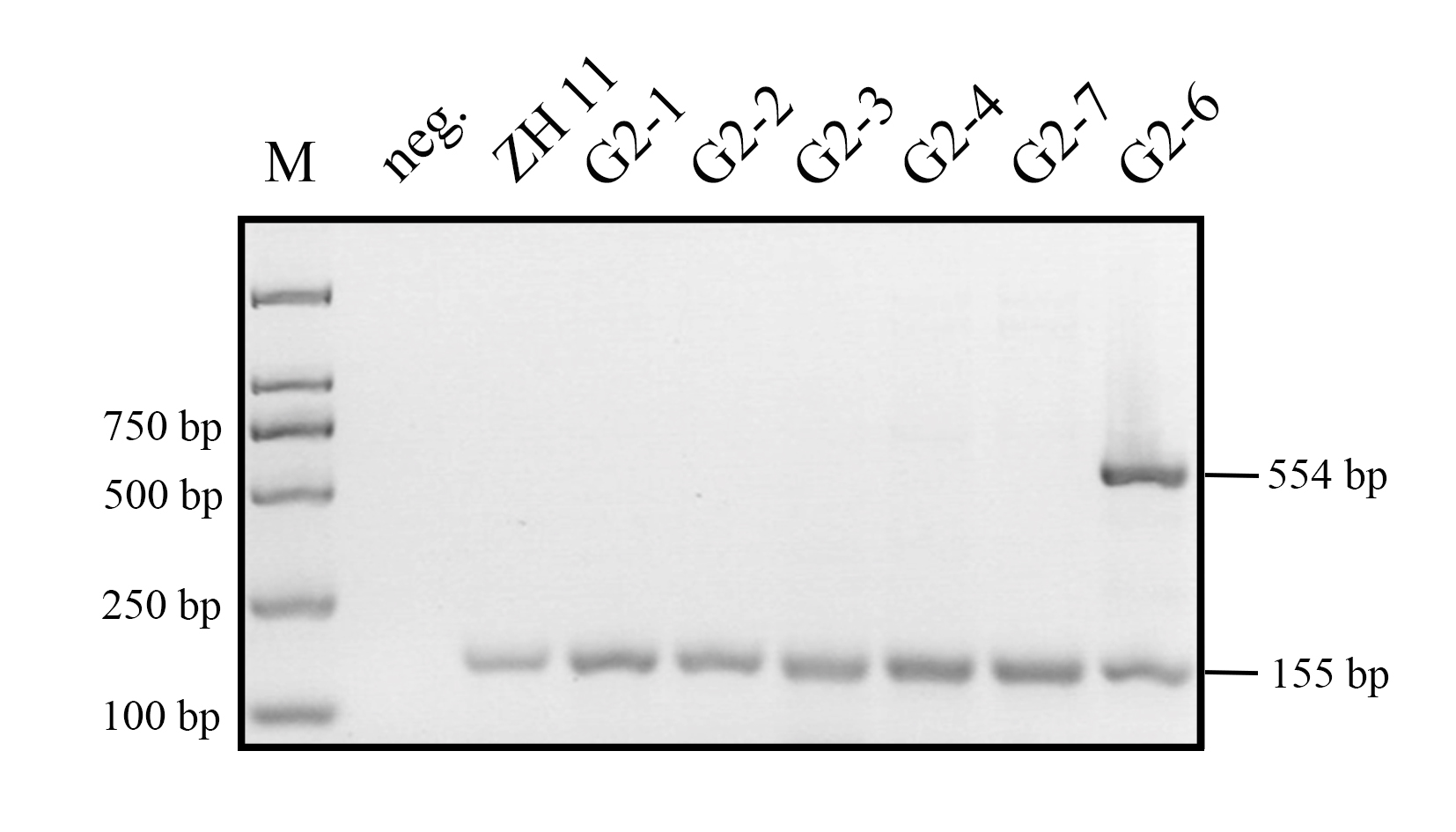

Supplement: FIGURE S2 — Integration-event-specific PCR of G2-6 transgenic lines. [file Image_2.JPEG]
