# Supplementary material for: Development and Event-specific Detection of Transgenic Glyphosate-resistant Rice Expressing the G2-EPSPS Gene
Source: Front Plant Sci. 2017 May 30;8:885. doi: 10.3389/fpls.2017.00885 (PMC5447670; doi:10.3389/fpls.2017.00885)
Supplement: Supplementary file 3 [file Table_1.docx]

Supplementary Table 1: Primers used for PCR analysis.

| Primer | Primer sequences (5'-3') |
| --- | --- |
| CTS-1 | GGATCCTATGGCCTCGATCTCTTCC |
| CTS-2 | CATCAGGCAAACACGCCATACTTCTTCAAACCCAATGGT |
| G2-1 | ACCATTGGGTTTGAAGAAGTATGGCGTGTTTGCCTGATGATG |
| G2-2 | GAGCTCTCAGTCGTTTAGGTGAACG |
| *Bam*H I-CTS-1 | GGATCCGGATCCTATGGCCTCGATCTCTTCC |
| *Sac* I-G2-2 | GAGCTCGAGCTCTCAGTCGTTTAGGTGAACG |
| G2-F | GACCAGGAGCCTTGTACCTTGAG |
| G2-R1 | GTCTTGAATGCGAATGCC |
| G2-R2 | CTGGCTGGACAAACCACCATC |
| GSP1 | ATTCCCAATACGAGGTCGCCAACATCT |
| GSP2 | GTGGTTGGCTTGTATGGAGCAGCAGAC |
| G2-OsF | CCGTATCGCTGTTCAAAACG |
| G2-GR | TTTCGATGATGCAGCTTGGG |
| SPSF | GCAACATCCAGCTCTACTCG |
| SPSR | GACGTCCATGAGCGAGTCC |
| OsUbq5F | ACCACTTCGACCGCCACTACT |
| OsUbq5R | ACGCCTAAGCCTGCTGGTT |
| G2-151F | ACCTTTGTGGTCACCAGCCAA |
| G2-151R | GTTTTTGCATGTACTCGTCGC |
